# Effects of Coconut Oil and Palm Oil on Growth, Rumen Microbiota, and Fatty Acid Profile of Suckling Calves

**DOI:** 10.3390/microorganisms11030655

**Published:** 2023-03-03

**Authors:** Fengming Hu, Minyu Piao, Chuntao Yang, Qiyu Diao, Yan Tu

**Affiliations:** 1 Institute of Feed Research, Chinese Academy of Agricultural Sciences, Beijing 100081, China; 2 Beijing Key Laboratory for Dairy Cow Nutrition, Beijing Municipal Commission of Science and Technology, Beijing 100081, China

**Keywords:** calf, fatty acids, rumen microbiota, liver, longissimus dorsi

## Abstract

This study aimed to evaluate the effects of coconut oil and palm oil in milk replacer (MR) on the growth performance, blood lipids, rumen fermentation, rumen microbiota, and fatty acid profile of hepatic and muscle of suckling calves. Thirty-six Holstein male calves were randomly assigned to three treatments. Three milk replacers containing different fat sources were as follows: control group (CON, milk fat), coconut oil group (CCO, coconut oil powder as fat), and palm oil group (PLO, palm oil powder as fat). Calves were weighed and blood sampled at 14, 28, 42, and 56 days old, respectively, and the feed intake and fecal score were recorded daily. Fat sources in milk replacers had no effects on body weight, ADG, DMI, fecal score, or days of abnormal fecal in suckling calves among the three groups, while the PLO group tended to decrease starter intake compared with the other groups. Serum concentrations of TC, HDL-C, LDL-C, and VLDL-C in the CCO group increased compared with those of the CON group. Palm oil also decreased the serum GLU concentration of calves but had no effects on serum lipids compared with milk fat. Coconut oil or palm oil had no effects on rumen fermentation, rumen chyme enzyme activity, rumen bacterial community richness and diversity, and dominant phyla and genera when compared with milk fat. However, compared with the CON group, the CCO group increased the proportion of MCFAs and n-6 PUFAs, and decreased the proportion of UFAs and MUFAs in liver tissue, while the PLO group increased the proportion of PUFAs and decreased the proportion of n-3 PUFAs in liver tissue. In addition, compared with the CON group, the CCO group increased the proportion of MCFAs, and decreased the proportion of UFAs and n-3 PUFAs in longissimus dorsi, while the PLO group increased the proportion of PUFAs and decreased the proportion of n-3 PUFAs in longissimus dorsi. In conclusion, compared with milk fat, coconut oil or palm oil in MR had no effects on growth performance, rumen fermentation, and rumen microflora but significantly increased serum lipids concentration and changed some proportions of MCFAs and PUFAs in liver and longissimus dorsi in suckling calves. These results indicate that coconut oil or palm oil as the sole fat source for MRs has no adverse effect on calf rumen fermentation and rumen microbiota but has a detrimental effect on n-3 PUFAs deposition in the liver and longissimus dorsi muscle.

## 1. Introduction

Milk fat is a natural and complex fat extracted from ruminant milk rich in fatty acids. It contains more than 400 fatty acids (FAs) [1], of which saturated fatty acids (SFAs) account for 65%, followed by monounsaturated fatty acids (MUFAs, 30%) and polyunsaturated fatty acids (PUFAs, 5%) [2]. Milk fat has long been a major source of fat for milk replacers (MRs) and a major ingredient in human infant food and dairy products. However, in order to meet the human demand for milk fat and reduce the cost of MR, it is necessary to study fat substitutes with abundant sources and low prices. Numerous studies have reported the effects of various animal fats or vegetable oils, such as lard, tallow, corn oil, soybean oil, coconut oil, or palm oil, on the performance and health of suckling calves [3,4,5]. Among them, coconut oil and palm oil are cheap vegetable oils derived from coconut and palm pulp or nuts, respectively, with abundant reserves in the world, and have been extensively studied as fat sources for MRs [5,6]. The replacement of milk fat in colostrum and milk with coconut oil significantly altered fat-soluble vitamins in the serum of neonatal calves, as first found by Rajaraman et al. [4]. In addition, more energy and fat in the body were deposited in calves that were fed greater amounts of medium-chain fatty acids (MCFAs) from coconut oil under cold stress conditions [6]. Bowen et al. further demonstrated that the use of coconut oil in milk replacers benefits calf health and performance based on skeletal growth and fecal score [7]. Similarly, Panahiha et al. observed that the combination of corn forage and palm oil fat in the starter created a positive interaction on the growth performance of suckling calves [8]. In addition, some studies have shown that the addition of some short-chain or medium-chain fatty acids such as sodium butyrate and medium-chain fatty acid monoglycerides to calf MRs has some potential benefits in the rumen development [9,10]. However, more studies focused on the effects of coconut oil or palm oil used in the starter on growth performance, health, ruminal fermentation, and blood metabolites [3,11]. There are limited reports in the literature on the effects of adding coconut oil or palm oil or their major fatty acids, MCFAs or palmitic acid, to MRs on rumen fermentation, rumen chymase activity, and rumen microflora in suckling calves. This may be related to the esophagus groove reflex of pre-ruminant calves. It can deliver liquid feed directly to the abomasum, and only about 3% of the liquid feed enters the rumen for fermentation [12]. However, some studies have indicated that on average, 12% to 20% of milk or MRs may leak into or backflow into the reticulorumen in suckling calves [13,14,15], which may affect rumen fermentation and rumen microflora, provide nutrients for the development of tissues and organs, such as the rumen, and improve the growth and development of the body. Therefore, it is necessary to conduct some research that focuses on the effects of coconut oil or palm oil formulated into MRs on rumen fermentation, rumen chymase activity, and rumen microorganisms. Therefore, we hypothesized that the usage of coconut oil or palm oil as a fat source for MRs would change rumen fermentation and rumen microflora in suckling calves. One of the objectives of this study was to investigate the effects of coconut oil and palm oil as a fat source for MRs on rumen fermentation, chymase activities, and rumen microbiota in suckling calves.

In addition, palm oil is rich in palmitic acid, followed by oleic acid and linoleic acid, which contain 50% SFAs, 40% MUFAs, and 10% PUFAs [16]. The fatty acid composition of coconut oil is different from that of milk fat and palm oil, as about 90% of the fatty acids are made of SFAs [17,18], and more than about 50% of the fatty acids are made of MCFAs, such as lauric acid [2]. Previous studies have shown that different dietary fats or fatty acids can affect the composition of fatty acids in the various body tissues of calves [19,20,21]. Jenkins et al. observed that high dietary intake of linoleic acid in corn oil or PUFAs in fish oil increases PUFAs concentration, such as linoleic acid, eicosapentaenoic acid, and eicosapentaenoic acid, in calf plasma, liver, platelets, muscle, and heart, respectively [21]. Garcia et al. [22] found that supplementation with linoleic acid to newborn calf MRs significantly increases the total percentage of n-6 and n-3 PUFAs in liver tissue. However, research on the effects of adding coconut oil or palm oil or their major fatty acids to MRs on the body fatty acid composition of dairy cows is still limited. We hypothesized that the use of coconut oil or palm oil as a fat source for MRs would change the fatty acid composition in the liver and muscle of suckling calves. Therefore, another purpose of this study is to evaluate the effects of coconut or palm oil on blood lipids and fatty acid composition in the liver and muscle of suckling calves. 

## 2. Materials and Methods

### 2.1. Animal Care

The experimental design and animal care and handling procedures were evaluated and approved by the Animal Ethics Committee of the Institute of Feed Research of the Chinese Academy of Agricultural Sciences (IFR-CAAS-20160715, Beijing, China).

### 2.2. Animals, Treatments, and Management

Thirty-six 3 ± 1 days old and healthy Holstein male calves were randomly selected for the experiment. All calves were fed colostrum and milk from 1 to 3 days after birth, and then fed fresh milk from 4 to 7 days old. Calves aged 7 days (42.8 ± 4.4 kg of body weight) were randomly assigned to one of the three treatments: (A) control group (CON), only whole milk powder as a fat source of MR (254 g/kg of crude protein (CP) and 127 g/kg of ether extract (EE); the levels of the main fatty acids including palmitic, oleic, and myristic acids were 4.15, 2.33, and 1.53 g/100 g MR, respectively); (B) coconut oil group (CCO), only coconut oil powder as a fat source of MR (259 g/kg of CP and 125 g/kg of EE; the levels of the main fatty acids including lauric and myristic acids were 4.90 and 2.00 g/100 g MR, respectively); (C) palm oil group (PLO), only palm oil powder as a fat source of MR (252 g/kg of CP and 122 g/kg of EE; the levels of the main fatty acids including palmitic and oleic acids were 6.72 and 4.19 g/100 g MR, respectively). Milk replacers were isonitrogenous and isocaloric at 250 g/kg of CP, 120 g/kg of EE, and 4.8 Mcal/kg metabolizable energy (ME) of DM. Nutrient levels and fatty acid composition of the MRs are presented in Supplementary Table S1. From 7 to 14 days old, calves were fed these MRs for an adaptation period of 1 week, then a 42-day trial was conducted. Calves were gradually weaned from 56 to 60 days old. The MRs were reconstituted to emulsions (12.5%, wet/vol) by cooling boiled water to 40 °C and then fed to calves on a DM basis at 10% of live weight twice a day (0700 and 1500 h) [23]. All calves were housed in individual hutches (1.6 × 3.6 m), fed using a bucket with a teat, and reared with ad libitum access to starter feed from 14 days old. The ingredient composition and nutrient profile of the starter are presented in Supplementary Table S2. The average ambient temperature was 9 °C (range −5~23 °C), and most of the time during the trial, it was at or below the lower critical temperature (LCT, 8~13 °C) [24,25] (Supplementary Figure S1).

### 2.3. Growth Performance and Feed Intake

The milk replacers and starter samples were collected weekly during the experiment to determine the content of dry matter (DM) (method 930.15), CP (method 990.03), EE (method 2003.05), ash (method 942.05), calcium (method 985.01), and phosphorus (method 985.01) according to AOAC International (2006) protocols [26]. Neutral detergent fiber (NDF) and acid detergent fiber (ADF) contents of the starter were determined according to the method of α-amylase [27].

The body weight of calves was measured before the morning feeding at 14, 28, 42, and 56 days old, respectively. The daily intake of the MRs and starter were recorded, and the total dry matter intake (DMI) and FAs intake of MR and starter for each calf were calculated. The Feed intake: Gain ratio (F: G ratio) was calculated as the ratio of total DMI to average daily gain [28]. Fecal scores were recorded several times daily by a blind test according to a 5-point score [29]: 1 = normal, thick in consistency, 2 = normally, but less thick, 3 = abnormally thin but not watery, 4 = watery, 5 = watery with abnormal coloring.

### 2.4. Blood Sampling and Analysis

Blood samples were obtained using jugular venipuncture at 14, 28, 42, and 56 days old and then centrifuged at 3000× *g* at 4 °C for 10 min. The obtained serum was transferred into 1.5 mL centrifuges tubes and stored at −20℃ before analysis. Glucose (GLU), cholesterol (TC), and triglyceride (TG) were measured using an automatic serum biochemical analyzer (KeHua, KHB-1280, Shanghai, China). High-density lipoprotein cholesterol (HDL-C), low-density lipoprotein cholesterol (LDL-C), very low-density lipoprotein cholesterol (VLDL-C), non-esterified fatty acids (NEFAs), and β-Hydroxybutyric acid (BHBA) were measured using bovine enzyme-linked immunosorbent assay kits (JianCheng Biotechnology Co., Ltd., Nanjing, China).

### 2.5. Ruminal Chyme Fermentation Parameters and Chyme Enzyme Activity

At 56 days old, ruminal chyme was collected through the mouth using an esophageal tube from six calves per treatment 2 h after the morning feeding as described by Paz et al. [30], and then it was divided into two tubes for microbial analysis and fermentation parameters and enzyme activities measurement, respectively. More specifically, the pH value and ammonia nitrogen (NH_3_-N) were measured using the methods of pH meter (206-pH2, Testo, Germany) and phenol-sodium hypochlorite colorimetric, respectively [31]. The volatile fatty acids (VFAs) were measured using a gas chromatograph (SP-3420A, Beijing Analytical Instrument Factory, Beijing, China) equipping a mega bore HP-MOLSIV column (film thickness: 30 m × 0.53 mm × 25 mm, Agilent Technologies, Palo Alto, CA, USA) [23]. The ruminal chyme enzyme activities including neutral protease, carboxymethylcellulose, α-amylase, xylanase, and lipases were measured as described previously [32].

### 2.6. Analysis of Microbiota

Total genome DNA from the ruminal content was extracted using the CTAB/SDS method, quantified, purified on 1% agarose gels, and finally diluted to 1 ng/μL using sterile water. Regions V4 of the bacterial 16S rRNA gene were amplified with specific primers (515F, GTGCCAGCMGCCGCGGTAA; 806R, GGACTACHVGGGTWTCTAAT). PCR products (400–450bp) were mixed in isodensity ratios. Then, the mixture of PCR products was purified with a Qiagen Gel Extraction Kit (Qiagen, Dusseldorf, Germany). Sequencing libraries were generated using TruSeq^®^ DNA PCR-Free Sample Preparation Kit (Illumina, CA, USA) following the manufacturer’s recommendations, and index codes were added. The library quality was assessed on the Qubit@ 2.0 Fluorometer (Thermo Scientific, Waltham, MA, USA) and Agilent Bioanalyzer 2100 system (Agilent Technologies, Palo Alto, CA, USA). Subsequently, the library was sequenced using an Illumina HiSeq2500 platform and 250 bp paired-end reads were generated.

### 2.7. Sequencing Data Analysis

Paired-end reads were merged using FLASH version 1.2.7 (http://ccb.jhu.edu/software/FLASH/), and the splicing sequences were called raw tags. The high-quality clean tags were obtained according to the QIIME (version 1.8.0, http://bio.cug.edu.cn/qiime/) quality-controlled process. The chimera sequences were determined and removed by comparison between tags and the reference database (Gold database, http://drive5.com/uchime/uchime_download.html) using the UCHIME algorithm. Sequence analysis was performed using UPARSE version 2.7.1. Sequences with ≥97% similarity were assigned to the same OTUs. A representative sequence for each OTU was screened for further annotation. For each representative sequence, the GreenGene Database (http://greengenes.lbl.gov/cgi-bin/nph-index.cgi) was used based on the RDP classifier version 2.2 (http://sourceforge.net/projects/rdp-classifier/) algorithm to annotate taxonomic information. Alpha diversity was applied in analyzing the complexity of species diversity for a sample using 4 indices, including Chao1, Shannon, Simpson, and ACE, with QIIME version 1.8.0 and displayed with R version 4.0.3. A principal component analysis (PCA) was used to calculate differences in the bacterial communities’ structure using R version 4.0.3.

### 2.8. Fatty Acid Profile Measurements

At 60 days old, six calves were selected and slaughtered from each group. Liver and muscle samples were collected and stored at −80 °C before analysis. Lipids in the liver and muscle were extracted according to the procedure of Folch et al. [33]. Fatty acid content was measured as described by Piao et al. [34]. Briefly, fatty acid was identified using an Agilent 7890A gas chromatography system (Agilent Technologies, Palo Alto, CA, USA) equipped with an automatic injector, a CP-sil88 capillary column (100 m × 0.25 mm × 0.2 μm, CP7489, J&W Science, MA, USA) and a hydrogen flame ion detector. 

### 2.9. Statistical Analysis

Data were analyzed using SAS 9.4 software (SAS Institute, Cary, NC, USA). The ADG, DMI, MR DMI, Starter DMI, F:G ratio, fecal score, abnormal fecal day, and serum parameters were analyzed using the MIXED procedure. The effects of treatment, day, and the interaction between treatments and day were included as fixed effects and the calf within treatments as a random effect. The fatty acids intake, liver tissue, and muscle tissue FA profile, rumen fermentation parameters, and rumen chyme activity were analyzed using a one-way ANOVA procedure. The domains among treatments were tested with the Wilcoxon rank-sum test. A *p*-value of <0.05 and 0.05 ≤ *p* < 0.10 were considered statistically significant and tendencies, respectively.

## 3. Results

### 3.1. Animal Growth Performance and Health

Except for the total DMI being affected by the interaction between treatment and day (*p* = 0.038; Supplementary Figure S2a), there was no interaction for any variates (*p* > 0.05). The intake of C6:0, C8:0, C10:0, C12:0, and C14:0 in the CCO group significantly increased (*p* < 0.05) when compared with those in the CON and PLO groups, while the intake of C14:1, C15:0, C16:0, C16:1, C18:0, C18:1, C18:2, C18:3, C20:0, C22:0, and C24:0 decreased significantly (*p* < 0.05) compared with the CON group. In addition, the intake of C16:0, C18:1, and C18:2 increased significantly in the PLO group (*p* < 0.05) when compared with the CON group, but the intake of C6:0, C8:0, C10:0, C12:0, and C14:0 obviously reduced (*p* < 0.05) compared with the CON and CCO groups (Table 1). The starter intake tended to decrease (*p* = 0.085) in the PLO group in comparison with the CON and CCO groups. However, the BW, ADG, DMI, and F:G ratio of suckling calves did not differ (*p* > 0.05) among the three groups. Moreover, the fecal scores and the average days of abnormal feces in the CCO and PLO groups had no significant difference (*p* > 0.05) compared with the CON group. 

### 3.2. Serum Parameters

The concentrations of BHBA and NEFA were affected by the interaction between treatment and day (*p* < 0.05; Supplementary Figure S2b,c), whereas these varieties were not influenced by the treatment effects (*p* > 0.05). The serum concentrations of TC, HDL-C, and LDL-C in the CCO group were higher (*p* < 0.05) than those in the CON and PLO groups (Table 2), and GLU in the CON group was higher (*p* < 0.05) in comparison with the PLO group. In addition, the serum level of TG in the CCO group was significantly higher (*p* < 0.05) when compared with the PLO group. 

### 3.3. Rumen Chyme Fermentation Parameters and Chyme Digestive Enzyme Activities

There was no significant difference in the rumen chyme fermentation parameters among the three groups (Supplementary Table S3). The rumen chyme enzyme activities of α-amylase, neutral protease, carboxymethyl cellulose, xylanase, and lipase were also not significantly affected by fatty acid supplementation in MR in suckling calves (Supplementary Table S4).

### 3.4. Community Structure and Composition of Rumen Microbiota

**Figure 1:**
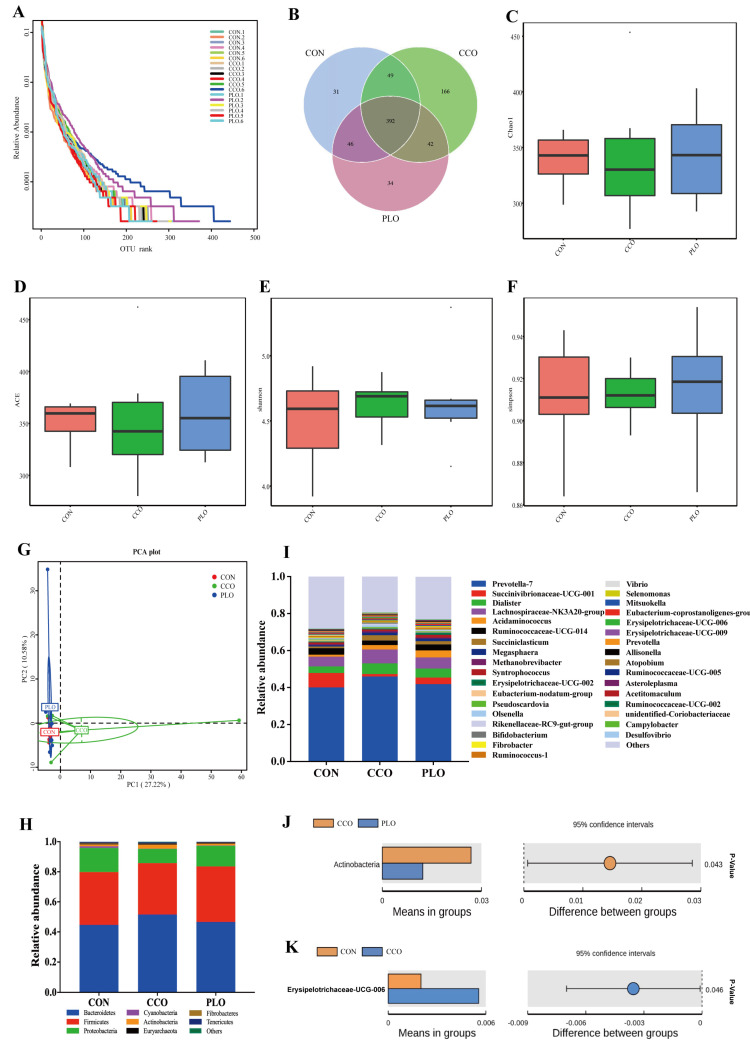
Rank abundance curves based on the OUT levels (**A**). Venn diagram of bacteria among the three groups (**B**). Bacterial richness and diversity among groups were estimated using the Chao1 (**C**), ACE (**D**), Shannon (**E**), and Simpson (**F**) indices. PCA analysis of rumen microtia among the three groups (**G**). The relative abundance of rumen microbiota at the phyla level (**I**). The relative abundance of rumen microbiota at the genera level (**H**). Comparison of microbial domains between CCO and PLO at the phyla level (**J**), and between CON and CCO at the genera level (**K**). Significantly different domains were tested with the Wilcoxon rank-sum test with an adjusted *p* value of <0.05.

A total of 1,371,853 ± 5646 clean tags were obtained from 18 rumen samples. The stable plateau based on the rank abundance curves of the OUT level suggested that the generated sequenced data were large enough in our study (Figure 1A). A quantity of 760 OUTs was attained based on a 97% of identification rate across all samples. The Venn diagram showed that 392 OUTs were shared among all samples, accounting for 51.5% of total OUTs, particularly, 518 OUTs in the CON group, 649 OUTs in the CCO group, and 514 OUTs in the PLO group (Figure 1B). Alpha diversity indices showed that rumen bacterial community richness (Chao1, AEC; Figure 1C,D) and diversity (Simpson, Shannon; Figure 1E,F) did not show any significant difference (*p* > 0.05) among the three groups. Similarly, PCA plots did not display obvious distance differences in bacterial structures in the three groups (Figure 1G). At the phyla level, the top five ruminal phyla bacteria were Bacteroidetes (47.5%), Firmicutes (35.4%), Proteobacteria (13.1%), Actinobacteria (1.7%), and Cyanobacteria (0.5%) across all samples, which accounted for greater than 98% of all phyla bacteria (Figure 1H). Actinobacteria in the CCO group had greater (*p* < 0.05) relative abundance than that in the PLO group (Figure 1J). In addition, at the genus level, the main genera across all samples were Prevotella_7 (42.7%), followed by Succinivibrionaceae_UCG-00, Dialister, Lachnospiraceae_NK3A20_group, Acidaminococcus, Ruminococcaceae_UCG-014, Succiniclasticum, Megasphaera, Methanobrevibacter, Syntrophococcus, which accounted for more than 68% of all genera bacteria (Figure 1I). However, those dominant genera had no significant differences (*p* > 0.05) among the three groups. Importantly, the relative abundance of Erysipelotrichaceae_UCG-006 was higher (*p* < 0.05) in the CCO group than that in the CON group (Figure 1K).

### 3.5. Fatty Acid Profile of Liver and Muscle Tissues

The concentrations of total fatty acid in the liver and longissimus dorsi were not different (*p* > 0.05) among the three groups (Table 3). In the liver tissue, the proportions of C14:0, C20:4, C24:1, MCFAs, and n-6 PUFAs in the CCO group were higher (*p* < 0.05) than those in the CON group, while the proportions of C18:1, C18:3, C20:1, C20:5, C22:6, UFAs, and MUFAs in the CCO group were lower than those in the CON group (*p* < 0.05). In addition, the proportions of C20:4, C24:1, and n-6 PUFAs in the PLO group were significantly higher (*p* < 0.05) in comparison with the CON group, while the proportions of C14:0, C16:1, C18:3, C20:1, C20:5, C22:6, and n-3 PUFA were lower (*p* < 0.05) when compared with the CON group; In addition, when compared with the PLO group, the proportions of C14:0, C20:5, and MCFAs in the CCO group were higher (*p* < 0.05), and the proportions of C24:1 and MUFAs were lower (*p* < 0.05). In the longissimus dorsi, the proportions of C14:0 and MCFAs in the CCO group were higher (*p* < 0.05) than those in the CON group, while the proportions of C18:0, C18:3, C20:1, C20:5, UFAs, and n-3 PUFAs in the CON group were higher (*p* < 0.05). The proportions of C20:4, C24:1, MUFAs, PUFAs, and n-6 PUFAs in the PLO group were higher (*p* < 0.05) than those in the CON group, while the proportions of C16:1, C17:0, C18:0, C18:3, C20:1, C20:5, and n-3 PUFAs in the CON group were higher (*p* < 0.05). Moreover, when compared with the PLO group, the proportions of C14:0, C16:1, C20:1, MCFAs, and n-3 PUFAs in the CCO group were higher (*p* < 0.05), and the proportions of C20:4, C24:1, UFAs, PUFAs, and n-6 PUFAs were lower (*p* < 0.05).

## 4. Discussion

Dietary fat is a crucial energy source for young calves in the functions of maintenance energy and growing energy requirements. Suckling calves, unlike mature animals, have about 3% body fat reserves, so excess energy must be provided for them to avoid negative consequences [35]. Previous studies showed that fat content, sources, and fatty acid composition in MRs could influence growth performance, health, gastrointestinal development, and immunity of suckling calves [9,25,29]. For example, Jaster et al. [36] demonstrated that additional fat addition into MRs could lead to an increase in ADG. On the contrary, some studies showed that the increase in fat content was only conducive to fat deposition, but it could cause negative effects on weight gain in suckling calves [37,38,39]. The decline in growth performance might mainly attribute to the increase in flow rates of total protein and fat out of the abomasum due to high fat (fat > 200 g/kg of DM) in MRs, and the decrease in the nutrient digestibility and starters intake [39,40]. Meanwhile, those benefits, including the improvement in the growth performance, development of gastrointestinal tract, immune response, and health in calves, resulting from adding functional fatty acids or essential fatty acids (EFAs) from flax oil [41], soybean oil [42], or porcine lard [43] into MRs, have been demonstrated by some researchers. For example, the supplementation with a blend of butyric acid, MCFAs, and linolenic acid into MRs could improve ADG and feed efficiency and reduce calf diarrhea [29]. In the present study, these parameters, such as growth performance, feed efficiency, and health of calves, did not show a significant difference between the CCO group rich in MCFAs and the PLO group rich in SFAs and MUFAs when compared with the CON group. These results differed from those studies as described above [41,42,43], which might be attributed to those differences in fat content and fatty acid composition. In this study, the intake of EFAs and some functional fatty acids such as butyric acid and α-linolenic acid in the three treatments was lower compared to the dose in other studies [41,42], which partly explains the lack of differences in growth performance and the health of suckling calves. In addition, no negative effects on starter intake were observed at the low-fat (120 g/kg DM) level, which was consistent with the results described by Hill et al. [39]. However, we observed that the PLO group tended to reduce starter intake. As far as we know, there is no relevant report about the reduction in young animals’ feed intake resulting from palm oil. Therefore, further research on the effect of feeding palm oil alone on feed intake is warranted.

The correlation between the concentrations of blood GLU, BHBA, and NEFA and the energy status of calves had been reported [44]. In the present study, the serum concentration of GLU in the PLO group decreased compared with the CCO and CON groups, which might be explained by the starter intake that tended to be lower in the PLO group than in the other groups. In the present study, the serum concentrations of TC, HDL-C, and LDL-C in the CCO group were higher than those in the PLO and CON groups. Compared with the CON group, the intake of SFAs increased in the CCO group (lactic acid 3.35 g/d vs. 30.71 g/d; myristic acid 9.32 g/d vs. 12.53 g/d) or the PLO group (palmitic acid 25.29 g/d vs. 40.86 g/d). However, diets with a high content of SFAs could increase the levels of blood cholesterol [45]. Those comparisons between SFAs diets (from coconut oil, palm oil, or lauric, myristic, and palmitic acid) and other UFAs diets showed that the levels of TC, HDL-C, and LDL-C increased in SFAs diets [16,46]. Similarly, in our study, those blood lipid concentrations were increased in the lauric and myristic acid-enriched CCO group when compared with the CON group. However, the serum concentration of cholesterol in the PLO group was similar to that in the CON group and lower than that in the CCO group, which was contrary to studies on palmitic acid/palm oil showing increasing blood cholesterol [47]. Similar to previous studies [48,49], total serum concentrations of LDL- and HDL-Cs in coconut oil rich in lauric acid increased when compared with palm oil rich in palmitic acid. These results may be related to the difference in fatty acid composition, the effects of lauric acid, palmitic acid, and oleic acid diets on serum total cholesterol concentration, and HDL-C that were sorted in order of lauric acid > palmitic acid > oleic acid [50].

Unlike mature ruminants, the rumen of calves in the first few weeks of life (until 4 weeks of life) has no significant fermentation function due to the esophageal groove reflex and low starter intake during the first 3 weeks of life [51]. For pre-ruminant calves, milk or milk replacer directly enters the abomasum through the esophageal groove reflex, and normally about 3% of milk or milk replacer leaks into the rumen for fermentation [12]. However, several studies indicated that some milk or milk replacer may link or backflow into the reticulorumen in dairy calves (0 to 25%) [13,14] and veal calves (18 to 35%) [15], which may affect the development of rumen fermentation, digestive enzyme activities, and microbiome. In addition, large amounts of milk entering the rumen could increase the risk of abnormal fermentation (acidosis) and clinical diseases (flatulence and diarrhea) in calves. In the present study, we observed that a coconut oil diet and palm oil diet did not affect the rumen fermentation, digestive enzyme activities, or alpha and beta diversity of bacteria. Similarly, previous studies showed that dietary fat sources (palm oil, soybean oil, tallow, flaxseed oil, or mixture oil) [3,52] and fatty acids composition (e.g., lauric acid, palmitic acid, linolenic acid) [11,53] did not affect ruminal pH, ammonia, or total VFAs concentrations in dairy calves. Moreover, palm oil rich in saturated fatty acids is generally used as rumen bypass fat because they do not have negative effects on rumen fermentation compared with unsaturated oils [54]. In contrast, more studies in vitro and in vivo showed that dietary fatty acids could change the rumen fermentation and rumen microorganisms [55,56]. Studies in dairy cows and calves showed that unsaturated fatty acids (e.g., linoleic acid, linolenic acid) have adverse effects on rumen microbiota and its fermentative activity compared with saturated fatty acids (palmitic acid) [57,58] probably due to more toxic of dietary PUFAs to rumen microbiota than SFAs. The PUFAs were converted into UFAs by hydrogenation of rumen microorganisms, and it would inhibit the growth of cellulolytic bacteria and some butyrate-producing bacteria, including the stearic acid producer *Clostridium proteoclasticum*, *Butyrivibrio hungatei*, and *Eubacterium ruminantium* [59]. In contrast, Jasem et al. reported that supplemental α-linolenic acid from flax oil increased the concentrations of total VFAs and proportion of butyrate in dairy calves [41], which could promote rumen epithelial cell proliferation in weaning calves [9]. Furthermore, numerous studies reported that coconut oil or lauric acid could change rumen protozoal numbers, microbial populations, and ruminal fermentation patterns in dairy cows and then affect nutrient digestion and methane emission [60,61,62]. However, Burdick et al. [63] reported that supplementation with MCFAs at 0.063% dietary DM in dairy cows decreases rumen bacterial richness, but it does not affect rumen fermentation. These results above might be related to fat content, fatty acid composition, fat addition methods, dietary nutrient level, rumen maturity, and cattle age. Rumen digestive enzymes (amylase, protease, xylanase, CMCase, and lipases) encoded and secreted by microbiota were associated with starch, fiber, and fatty acid metabolism [64,65,66]. Yabuuchi et al. [66] observed that supplemental coconut oil rich in lauric acid increase amylase activity in vitro rumen fermentation. Hristov et al. [67] observed that supplementation with sodium laurate could reduce CMCase and xylanase activities of ruminal contents in dairy cows compared to the control group. The effects of coconut oil or lauric acid on rumen digestive enzymes may be related to the effect on rumen microorganisms. Liu et al. [68] found that supplementation with glycerol monolaurate or the combination of glycerol monolaurate and tributyrin increased the relative abundance of *Actinobacteria* and *Verrucomicrobia* and decreased the relative abundance of *Ruminococcus*. However, the digestive enzyme activities of the rumen contents did not show differences between coconut oil rich in lauric acid or palm oil rich in palmitic acid and the CON group, which was consistent with the results of rumen microbial diversity and relative abundance. In this study, the *Firmicutes*, *Bacteroidetes,* and *Proteobacteria* dominated the rumen by accounting for ~95% at the phyla level, which was similar to the dominant rumen flora reported by Castro et al. [51] in dairy calves. Interestingly, the relative abundance of *Actinobacteria* in the CCO group increased at the phyla level compared with the PLO group, and its main function was responsible for the production of a variety of antibiotics, such as streptomycin, oxytetracycline, tetracycline, gentamicin, etc. Moreover, the relative abundance of unclassified members of *Erysipelotrichaceae* (*Erysipelotrichaceae_UCG-006*) at the genus level in the CCO group increased compared with the milk fat group. *Erysipelotrichaceae* in the rumen has been reported to have a strong negative correlation with methane emissions [69]. These results showed that coconut oil has a potential benefit on the development of rumen microflora and reducing methane emissions.

Previous studies showed that the fatty acid esterification and triacylglycerol accumulation in the hepatic cells increase in pre-ruminant calves that are fed MRs containing coconut oil rich in MCFAs compared to those of calves fed beef tallow rich in SFAs and MUFAs [70]. However, some studies observed that calves fed MRs containing coconut oil or lard fat did not show an increase the lipids infiltration in the liver compared with calves fed whole milk [6]. Similarly, the proportion of total fatty acid in the liver and longissimus dorsi of calves in the CCO group or the PLO group did not show significant changes compared with those in the CON group. This might be attributed to the fact that calves were in a cold environment for a long time (supplementary Figure S1) and the dietary protein and fat levels are relatively low in this trail, so calves need a lot of fat oxidation for energy to meet the additional energy requirement. Thus, the effect of coconut oil and palm oil on calf liver deposition under high protein and high-fat conditions need further research. In the present study, we found that coconut oil increased the intake of MCFAs and n-6 PUFAs and decreased the intake of MUFAs and n-3 PUFAs compared with milk fat. Compared with milk fat, palm oil increased the intake of SFAs, MUFAs, and n-6 PUFAs and decreased the intake of MCFAs and n-3 PUFAs. Consistent with this, coconut oil increased the proportion of MCFAs in the longissimus dorsi and the proportion of MCFAs and n-6 PUFAs in the liver and decreased the proportion of UFAs and n-3 PUFAs in the longissimus dorsi and the proportion of UFAs, MUFAs, and n-3 PUFAs in the liver. Palm oil increased the proportion of n-6 PUFAs in the liver and longissimus dorsi and decreased the proportion of C14:0 and n-3 PUFAs in the liver and the proportion of MUFAs and n-3 PUFAs in longissimus dorsi. However, although the percentage of MCFAs in the liver and longissimus dorsi increased in the coconut oil group compared with the milk fat group, the sum of the percentage of MCFAs accounted for little in the liver and longissimus dorsi. The reason might be related to the oxidative rate and metabolic pathway of varying FAs in the liver. Unlike LCFAs, MCFAs could be rapidly absorbed from the gut via a portal vein into the liver, and they are mostly used for oxidation in the liver cells [17,18]. Therefore, the decreased percentage of long-chain saturated and unsaturated FAs might be related to most of the FAs absorbed into the liver being directly oxidized and few FAs acting as substrates for *de novo* synthesis in the CCO group compared with the CON group. In addition, the dominant fatty acid was stearic (C18:0), followed by oleic (C18:1), linoleic (C18:2), palmitic (C16:0), behenic (C22:0), and arachidonic acid (C20:4) in the liver, and the dominant fatty acid was oleic (C18:1), followed by linoleic (C18:2), palmitic acid (C16:0), stearic (C18:0), arachidonic (C20:4), and behenic acid (C22:0) in the longissimus dorsi. This indicated that the fat source did not significantly change the proportion of major fatty acids in the liver or longissimus dorsi. As far as we know, there has been no study to report that dietary coconut oil changes the percentage of FAs in the liver or longissimus dorsi of suckling calves. Thus, a future study is warranted to elucidate the mechanisms of coconut oil or palm oil causing the difference in fatty acid composition in the liver and longissimus dorsi.

## 5. Conclusions

Coconut oil or palm oil replacements for milk fat in MRs had no effects on growth, health, rumen fermentation and enzyme activity, or rumen microbial abundance and diversity. Compared with milk fat, palm oil decreased blood glucose concentration, and coconut oil increased some blood lipid concentrations. Moreover, coconut oil and palm oil had no effects on the level of total fatty acids but significantly changed some proportions of MCFAs and PUFAs in the liver and longissimus dorsi in suckling calves.

## Figures and Tables

**Table 1:** Effects of coconut oil or palm oil in MRs on growth performance and health of suckling calves.

Items	Treatments			SEM	*p*-Value		
	CON	CCO	PLO		T	D	T × D
BW at day 14, kg	43.6	44.9	44.3	0.85	0.830	-	-
BW at day 56, kg	74.8	75.8	71.4	1.72	0.460	-	-
ADG, kg/d	0.74	0.73	0.66	0.047	0.326	<0.01	0.177
DMI ^1^, kg/d	1.18	1.20	1.11	0.043	0.288	<0.01	0.038
MR intake, kg/d	0.61	0.63	0.61	0.030	0.786	<0.01	0.494
Starter intake, kg/d	0.57	0.57	0.52	0.028	0.085	<0.01	0.623
F:G ratio ^2^	1.78	1.79	1.92	0.146	0.609	<0.01	0.282
Fecal score ^3^	2.07	2.01	2.03	0.091	0.815	<0.01	0.987
Abnormal, d/week	0.98	0.94	0.88	0.396	0.966	<0.01	0.473
Fatty acid intake, g/d							
Caproic acid, C6:0	0.98 ^a^	0.38 ^b^	0.06 ^c^	0.076	<0.01	-	-
Caprylic acid, C8:0	0.67 ^b^	4.26 ^a^	0.00 ^c^	0.358	<0.01	-	-
Capric acid, C10:0	1.95 ^b^	3.76 ^a^	0.06 ^c^	0.297	<0.01	-	-
Lauric acid, C12:0	3.35 ^b^	30.71 ^a^	0.24 ^c^	2.616	<0.01	-	-
Myristic acid, C14:0	9.32 ^b^	12.53 ^a^	1.03 ^c^	0.969	<0.01	-	-
Myristolic acid, C14:1	0.85 ^a^	0.00 ^b^	0.00 ^b^	0.079	<0.01		
Pentadecanoic acid, C15:0	1.04 ^a^	0.00 ^b^	0.06 ^b^	0.093	<0.01		
Palmitic acid, C16:0	25.29 ^b^	7.65 ^c^	40.86 ^a^	2.720	<0.01	-	-
Palmitoleic acid, C16:1	1.22 ^a^	0.00 ^c^	0.12 ^b^	0.108	<0.01		
Stearic acid, C18:0	8.11 ^a^	2.57 ^b^	4.50 ^c^	0.453	<0.01	-	-
Oleic acid, C18:1	14.20 ^b^	5.95 ^c^	25.47 ^a^	1.620	<0.01	-	-
Linoleic acid, C18:2 n-6	0.61 ^c^	1.44 ^b^	5.96 ^a^	0.482	<0.01	-	-
α-linolenic acid, C18:3 n-3	0.55 ^a^	0.00 ^c^	0.12 ^b^	0.046	<0.01	-	-
Arachidic acid, C20:0	0.12 ^b^	0.06 ^c^	0.37 ^a^	0.027	<0.01	-	-
Eicosadienoic acid, C20:1	0.06 ^b^	0.06 ^b^	0.12 ^a^	0.006	<0.01	-	-
Behenic acid, C22:0	0.12 ^a^	0.00 ^c^	0.06 ^b^	0.010	<0.01	-	-
Lignoceric acid, C24:0	0.18 ^a^	0.06 ^c^	0.12 ^b^	0.010	<0.01	-	-

^1^ DMI = Starter intake + MR intake. ^2^ F:G ratio = feed-to-gain ratio, calculated as the ratio of total DMI to ADG. ^3^ Fecal scores were recorded daily according to a 5-point score: 1 = normal, thick in consistency, 2 = normally, but less thick, 3 = abnormally thin but not watery, 4 = watery, 5 = watery with abnormal coloring. ^a,b,c^ Values within a row with different superscripts differ significantly at *p* < 0.05.

**Table 2:** Effects of coconut oil or palm oil in MRs on serum parameters of suckling calves.

Items ^1^	Treatments			SEM	*p*-Value		
	CON	CCO	PLO		T	D	T × D
GLU, mmol/L	4.04 ^a^	4.06 ^a^	3.44 ^b^	0.178	0.007	0.001	0.589
BHBA, mmol/L	0.74	0.89	0.72	0.106	0.131	0.013	0.008
TC, mmol/L	1.78 ^b^	2.43 ^a^	1.62 ^b^	0.179	0.002	0.304	0.267
TG, mmol/L	0.15 ^ab^	0.18 ^a^	0.12 ^b^	0.018	0.019	0.006	0.179
HDL-C, mmol/L	0.61 ^b^	0.79 ^a^	0.55 ^b^	0.054	0.003	0.822	0.823
LDL-C, mmol/L	1.10 ^b^	1.55 ^a^	1.08 ^b^	0.101	0.007	0.493	0.529
VLDL-C, mmol/L	0.38	0.40	0.35	0.058	0.278	0.319	0.281
NEFA, μmol/L	169.3	171.9	158.8	16.53	0.278	0.015	0.013

^1^ GLU = glucose; TC = cholesterol; TG = triglyceride; HDL-C = high-density lipoprotein cholesterol; LDL-C = low-density lipoprotein cholesterol; VLDL-C = very low-density lipoprotein cholesterol; NEFAs = non-esterified fatty acids; BHBA = β-Hydroxybutyric acid. ^a,b^ Values within a row with different superscripts differ significantly at *p* < 0.05.

**Table 3:** Effects of coconut oil or palm oil in MRs on liver and longissimus dorsi fatty acid composition of suckling calves.

Items	Tissue	Treatment			SEM	*p*-Value
		CON	CCO	PLO		
Total fatty acid, %	Liver	13.98	14.21	14.13	0.987	0.406
	Longissimus dorsi	1.12	1.09	1.25	0.075	0.436
Fatty acid profile, % of total fatty acid						
Butyric acid, C4:0	Liver	0.22	0.23	0.25	0.015	0.186
	Longissimus dorsi	0.26	0.23	0.31	0.016	0.126
Caproic acid, C6:0	Liver	0.37	0.38	0.42	0.131	0.218
	Longissimus dorsi	0.85	0.77	0.93	0.037	0.202
Myristic acid, C14:0	Liver	0.82 ^b^	1.62 ^a^	0.25 ^c^	0.155	<0.01
	Longissimus dorsi	0.71 ^b^	2.37 ^a^	0.34 ^b^	0.271	<0.01
Palmitic acid, C16:0	Liver	13.06	13.30	12.70	0.364	0.364
	Longissimus dorsi	16.48	16.29	16.93	0.373	0.848
Palmitoleic acid, C16:1	Liver	0.60 ^a^	0.42 ^ab^	0.22 ^b^	0.057	0.010
	Longissimus dorsi	1.19 ^a^	1.26 ^a^	0.75 ^b^	0.076	0.004
Heptadecanoic acid, C17:0	Liver	1.59	1.49	1.50	0.016	0.129
	Longissimus dorsi	0.85 ^a^	0.75 ^ab^	0.68 ^b^	0.067	0.043
Stearic acid, C18:0	Liver	22.58	21.40	22.52	0.341	0.297
	Longissimus dorsi	13.84 ^a^	12.32 ^b^	12.79 ^b^	0.222	0.007
Oleic acid, C18:1	Liver	15.27 ^a^	13.39 ^b^	14.32 ^ab^	0.359	0.021
	Longissimus dorsi	24.89	23.32	22.49	0.509	0.154
Linoleic acid, C18:2 n-6	Liver	15.08	15.62	15.50	0.332	0.345
	Longissimus dorsi	20.21	20.59	22.62	0.689	0.203
α-linolenic acid, C18:3 n-3	Liver	0.62 ^a^	0.18 ^b^	0.20 ^b^	0.070	<0.01
	Longissimus dorsi	0.53 ^a^	0.09 ^b^	0.07 ^b^	0.059	<0.01
Eicosenoic acid, C20:1	Liver	1.04 ^a^	0.20 ^b^	0.01 ^b^	0.126	<0.01
	Longissimus dorsi	1.71 ^a^	1.31 ^b^	0.66 ^c^	0.117	<0.01
Eicosadienoic acid, C20:2 n-6	Liver	0.65	0.64	0.65	0.054	0.516
	Longissimus dorsi	-	-	-	-	-
Arachidonic acid, C20:4 n-6	Liver	9.76 ^b^	10.89 ^a^	11.40 ^a^	0.450	<0.01
	Longissimus dorsi	9.01 ^b^	9.95 ^b^	11.33 ^a^	0.354	0.003
Eicosapentaenoic acid, C20:5 n-3(EPA)	Liver	1.35 ^a^	0.66 ^b^	0.09 ^c^	0.146	<0.01
	Longissimus dorsi	1.21 ^a^	0.65 ^b^	0.42 ^b^	0.095	<0.01
Docosahexaenoic acid, C22:6 n-3(DHA)	Liver	2.24 ^a^	1.62 ^b^	1.70 ^b^	0.119	0.041
	Longissimus dorsi	-	-	-	-	-
Behenic acid, C22:0	Liver	13.06	14.25	14.35	0.618	0.186
	Longissimus dorsi	5.70	6.85	6.12	0.215	0.080
Nervonic acid, C24:1	Liver	1.59 ^c^	2.75 ^b^	3.73 ^a^	0.247	<0.01
	Longissimus dorsi	1.72 ^b^	1.81 ^b^	2.45 ^a^	0.102	<0.01
Others ^1^	Liver	0.10	0.96	0.19	-	-
	Longissimus dorsi	0.84	1.44	1.11	-	-
MCFAs	Liver	0.37 ^b^	0.56 ^a^	0.42 ^b^	0.029	0.010
	Longissimus dorsi	0.90 ^b^	1.46 ^a^	0.93 ^b^	0.085	0.001
SFAs	Liver	51.80	52.72	52.11	0.603	0.156
	Longissimus dorsi	38.78	39.92	38.30	0.586	0.171
UFAs	Liver	48.20 ^a^	46.37 ^b^	47.83 ^ab^	0.621	0.034
	Longissimus dorsi	60.48 ^a^	58.98 ^b^	60.79 ^a^	0.435	0.046
MUFAs	Liver	18.52 ^a^	16.76 ^b^	18.28 ^a^	0.246	0.049
	Longissimus dorsi	29.50 ^a^	27.73 ^ab^	26.36 ^b^	0.528	0.041
PUFAs	Liver	29.70	29.61	29.54	0.448	0.128
	Longissimus dorsi	30.96 ^b^	31.28 ^b^	34.44 ^a^	0.679	0.046
n-3 PUFAs	Liver	4.21 ^a^	2.46 ^b^	1.99 ^b^	0.287	<0.01
	Longissimus dorsi	1.75 ^a^	0.75 ^b^	0.49 ^c^	0.145	<0.01
n-6 PUFAs	Liver	25.49 ^b^	27.15 ^a^	27.55 ^a^	0.509	<0.01
	Longissimus dorsi	29.22 ^b^	30.54 ^b^	33.95 ^a^	0.702	0.022

^1^ Others means the sum of all fatty acids less than 0.1% in all three treatments. MCFAs = total of medium-chain fatty acids (6- to 12-carbon FAs); SFAs = total of saturated fatty acids; MUFAs = total of monounsaturated fatty acids; PUFAs = total of polyunsaturated fatty acids. ^a,b,c^ Values within a row with different superscripts differ significantly at *p* < 0.05.

## Data Availability

The datasets used and analyzed during the current study are available from the corresponding author (Y.T.) on reasonable request.

## Supplementary Materials

The following supporting information can be downloaded at: https://www.mdpi.com/article/10.3390/microorganisms11030655/s1, Supplementary Table S1: Nutrition levels and fatty acid composition of milk replacers; Table S2: The ingredient composition and nutrient profile of the starter; Table S3: Effect of medium-chain fatty acids and palmitic acid supplementation on fermentation parameters of suckling calves; Table S4: Effect of medium-chain fatty acids and palmitic acid supplementation on enzyme activity of suckling calves (per gram of true protein); Figure S1: The maximum (Max), average (Ave), and minimum (Min) ambient temperature the daily during the trial.
