# Supplementary material for: Effects of Coconut Oil and Palm Oil on Growth, Rumen Microbiota, and Fatty Acid Profile of Suckling Calves
Source: Microorganisms. 2023 Mar 3;11(3):655. doi: 10.3390/microorganisms11030655 (PMC10057803; doi:10.3390/microorganisms11030655)
Supplement: Supplementary file 1 [file microorganisms-11-00655-s001.zip › microorganisms-2253525-supplementary.pdf]

**Table S1** Nutrition levels, and fatty acid composition of MRs

| Items | MR[1] | | |
|---|---|---|---|
| | CON | CCO | PLO |
| Nutrition, g/kg DM[2] | | | |
| CP | 254 | 259 | 252 |
| EE | 127 | 125 | 122 |
| Ash | 64 | 63 | 64 |
| Ca | 14 | 14 | 14 |
| P | 13 | 13 | 13 |
| ME, Mcal/kg | 4.82 | 4.80 | 4.87 |
| Main fatty acids, g/100g MR[3] | | | |
| C12:0 | 0.55 | 4.90 | 0.04 |
| C14:0 | 1.53 | 2.00 | 0.17 |
| C16:0 | 4.15 | 1.22 | 6.72 |
| C18:0 | 1.33 | 0.41 | 0.74 |
| C18:1 | 2.33 | 0.95 | 4.19 |
| C18:2, n-6 | 0.10 | 0.23 | 0.98 |
| MCFAs | 1.14 | 6.24 | 0.06 |
| LCFAs | 10.13 | 4.84 | 12.94 |
| SFAs | 2.88 | 1.19 | 5.22 |
| UFAs | 7.84 | 4.99 | 7.74 |
| MUFAs | 2.68 | 0.96 | 4.23 |
| PUFAs | 0.19 | 0.23 | 1.00 |

[1] MR=milk replacer; CON, only whole milk powder as fat source of MR; CCO, only coconut oil powder as fat sources of MR; PLO, only palm oil powder as fat sources of MR.

[2] DM = dry matter; CP = crude protein; EE = ether extract; NDF = neutral detergent fiber; ADF = acid detergent; Ca = calcium; P = total phosphate.

[3] MCFAs = total of medium-chain fatty acids (6- to 12-carbon FA); LCFAs = total of long-chain fat-ty acids (>12-carbon FA), SFAs= total of saturated fatty acids; UFAs = total of unsaturated fatty acids, MUFAs=total of monounsaturated fatty acids; PUFAs=total of polyunsaturated fatty acids.

**Table S2** The ingredient composition and nutrient profile of starter

| **Ingredients,** g/kg | **Content** | **Nutrients** | **Content** |
|---|---|---|---|
| Extrude corn | 100 | CP, g/kg DM | 236.3 |
| Wheat flour | 45 | EE, g/kg DM | 49.8 |
| Soybean meal | 250 | NDF, g/kg DM | 165.6 |
| Extruded soybean | 80 | ADF, g/kg DM | 67.8 |
| Wheat bran | 60 | Ash, g/kg DM | 76.9 |
| Soybean molasses | 70 | Ca, g/kg DM | 15.1 |
| DDGS | 50 | P, g/kg DM | 9.6 |
| Glucose | 20 | ME,[2] Mcal/kg DM | 4.50 |
| Dried whey powder | 30 | | |
| Limestone | 22 | | |
| $CaHPO_4$ | 5 | | |
| NaCl | 8 | | |
| Premix [1] | 10 | | |

[1] The premix provided the following per kg of the starter: vitamin A 15 000 IU, vitamin D 5 000 IU, vitamin E 50 mg, Fe 90 mg, Cu 12.5 mg, Mn 60 mg, Zn 100 mg, Se 0.3 mg, I 1.0 mg, Co 0.5 mg.

[2] Calculated according to NRC (2001).

**Table S3** Effect of coconut oil or palm oil in MRs on rumen fermentation parameters of suckling calves

| Items [1] | Treatment | | | SEM | *P*-value |
|---|---|---|---|---|---|
| | CON | CCO | PLO | | |
| pH | 6.60 | 6.31 | 6.39 | 0.082 | 0.349 |
| $NH_3$-N, mg/L | 72.9 | 73.8 | 74.1 | 0.856 | 0.871 |
| Total VFA, mM | 81.6 | 84.7 | 84.4 | 3.275 | 0.691 |
| Acetate, % | 54.99 | 51.08 | 52.46 | 1.178 | 0.454 |
| Propionate, % | 28.55 | 31.46 | 31.38 | 1.425 | 0.688 |
| Isobutyric, % | 1.55 | 1.74 | 1.49 | 0.149 | 0.798 |
| Butyrate, % | 7.67 | 7.36 | 7.33 | 0.458 | 0.956 |
| Isovaleric, % | 3.27 | 3.95 | 3.26 | 1.309 | 0.723 |
| Valerate, /% | 3.97 | 4.41 | 4.08 | 0.155 | 0.560 |
| Acetate/Propionate ratio | 1.98 | 1.70 | 1.71 | 0.112 | 0.562 |

[1] $NH_3$-N = ammonia; Total VFA = total volatile fatty acid.

**Table S4** Effect of coconut oil or palm oil in MRs on rumen digestive enzyme activities of suckling calves (per gram of true protein)

| Items [1] | Treatments | | | SEM | *P*-value |
|---|---|---|---|---|---|
| | CON | CCO | PLO | | |
| $\alpha$-amylase, U/g | 40.3 | 41.9 | 45.7 | 2.903 | 0.753 |
| Neutral protease, U/g | 75.0 | 75.6 | 80.2 | 1.804 | 0.459 |
| CMCase, U/g | 8.3 | 8.4 | 8.4 | 0.182 | 0.931 |
| Xylanase, U/g | 17.6 | 17.2 | 17.2 | 0.631 | 0.959 |
| Lipases, U/g | 15.3 | 15.6 | 15.1 | 0.453 | 0.876 |

[1] CMCase = carboxymethyl cellulose.

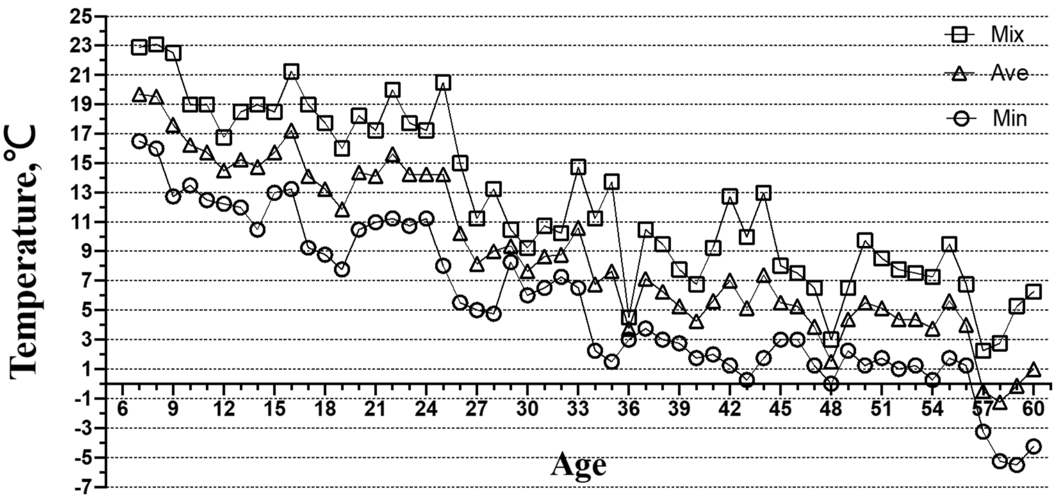


**Figure S1.** The maximum (Max), average (Ave), and minimum (Min) ambient temperature the daily during the trial. The average temperature of 9°C, ranging from –5 ~ 23°C.

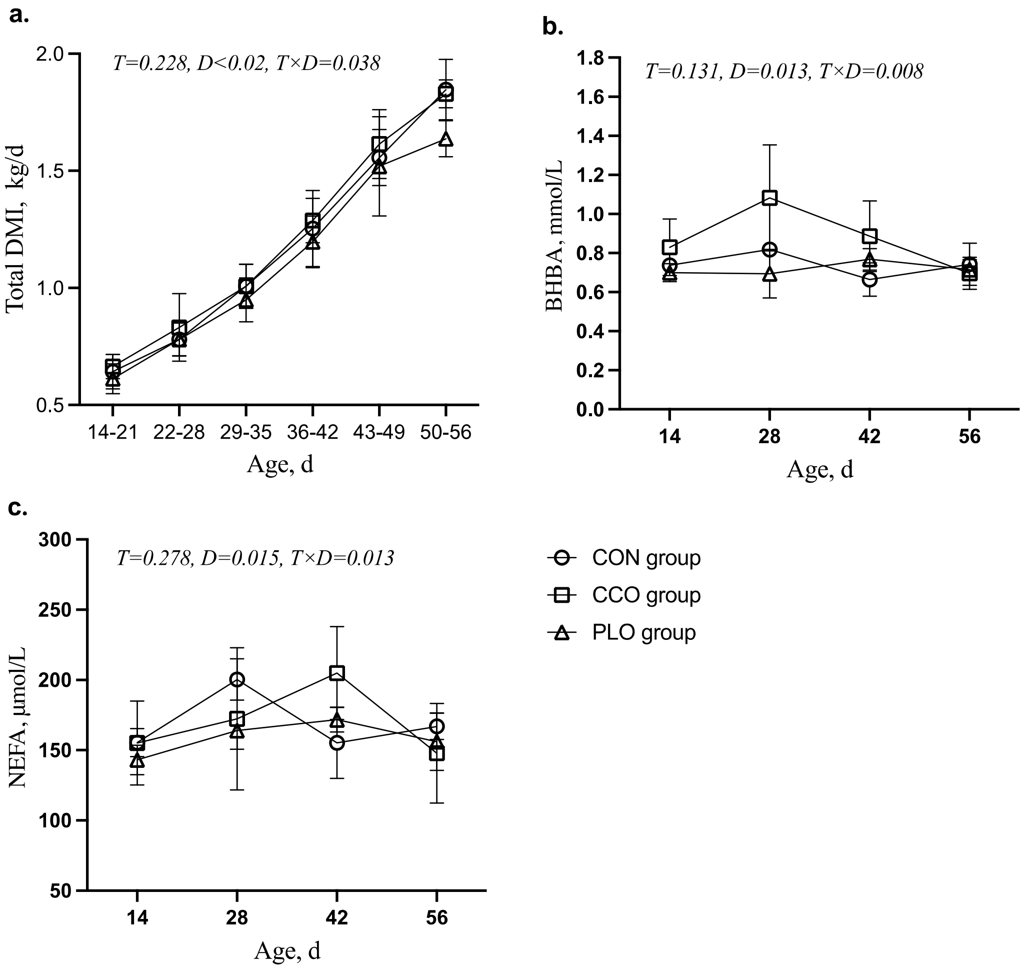


**Figure S2.** **a**. Effects of different sources of fat on total DMI in suckling calves; **b**. Effect of different sources of fat on concentrations of serum BHBA in suckling calves; **c**. Effect of different sources of fat on concentrations of serum NEFA in suckling calves; T= *p*-value of treatment, D= *p*-value of age, T × D= *p*-value of interaction between treatment and age
